# *STAT3* Single Nucleotide Polymorphism rs4796793 SNP Does Not Correlate with Response to Adjuvant IFNα Therapy in Stage III Melanoma Patients

**DOI:** 10.3389/fmed.2014.00047

**Published:** 2014-11-28

**Authors:** David Schrama, Selma Ugurel, Antje Sucker, Cathrin Ritter, Marc Zapatka, Dirk Schadendorf, Jürgen Christian Becker

**Affiliations:** ^1^Department of Dermatology, University Hospital Würzburg, Würzburg, Germany; ^2^Department of Dermatology, University Hospital Essen, Essen, Germany; ^3^Department of Dermatology, Medical University of Graz, Graz, Austria; ^4^Department of Translational Skin Cancer Research, German Cancer Research Center, Heidelberg, Germany; ^5^Department of Molecular Genetics, German Cancer Research Center, Heidelberg, Germany

**Keywords:** melanoma, interferon, predictive marker, STAT3, single nucleotide polymorphism

## Abstract

Interferon alpha (IFNα) is approved for adjuvant treatment of stage III melanoma in Europe and the US. Its clinical efficacy, however, is restricted to a subpopulation of patients while side effects occur in most of treated patients. Thus, the identification of predictive biomarkers would be highly beneficial to improve the benefit to risk ratio. In this regard, STAT3 is important for signaling of the IFNα receptor. Moreover, the *STAT3* single-nucleotide polymorphism (SNP) rs4796793 has recently been reported to be associated with IFNα sensitivity in metastatic renal cell carcinoma. To translate this notion to melanoma, we scrutinized the impact of rs4796793 functionally and clinically in this cancer. Interestingly, melanoma cells carrying the minor allele of rs4796793 were the most sensitive to IFNα *in vitro*. However, we did not detect a correlation between SNP genotype and *STAT3* mRNA expression for either melanoma cells or for peripheral blood lymphocytes. Next, we analyzed the impact of rs4796793 on the clinical outcome of 259 stage III melanoma patients of which one-third had received adjuvant IFNα treatment. These analyses did not reveal a significant association between the *STAT3* rs4796793 SNP and patients’ progression free or overall survival when IFNα treated and untreated patients were compared. In conclusion, *STAT3* rs4796793 SNP is no predictive marker for the efficacy of adjuvant IFNα treatment in melanoma patients.

## Introduction

Malignant melanoma is an aggressive skin cancer originating from melanocytes. Due to the continuously raising incidence of melanoma among the Caucasian population, it represents an increasing health problem. Indeed, in the US, melanoma is one of the few common cancers with increasing incidence rates over the last decade, i.e., a 2.4% increase per year among white women and 2.1% among white men during 1999–2009 (1). A total of 68,130 new cases of melanoma, and 8,700 patients dying from melanoma had been predicted for 2010 in the US (2). Moreover, the economic burden of this disease is also reflected by the recent report that an individual in the US loses on average 20.4 years of their potential lifetime as a result of melanoma mortality compared with 16.6 years for all other malignant cancers (3).

The prognosis of a melanoma patient is largely dependent on the stage of disease. While early-stage melanoma can be cured in most cases by surgical excision of the tumor, already for patients with loco-regional disease beyond the primary tumor the prognosis is highly impaired. Among patients with nodal metastases, the 5-year survival rates were 78, 59, and 40% for patients with stage IIIA, IIIB, and IIIC melanoma, respectively. The sub-grouping is based on primary tumor characteristics, ulceration of the primary tumor, loco-regional spreading, and number of affected lymph nodes (4). This impaired prognosis is despite the initial curative attempt of radical surgery to remove the primary tumor and loco-regional metastases (4).

Based on the high risk of relapse of stage III melanoma patients, which by surgical interventions are rendered to no evidence of disease (NED), adjuvant therapy would be indicated. The only currently approved adjuvant therapy – despite a recently reported positive clinical trial for adjuvant ipilimumab therapy of stage III melanoma – is interferon-α (IFNα). In 1995, high-dose IFNα-2b, and in 2011, pegylated IFNα-2b has been approved by the FDA for melanoma patients who are at high risk of recurrence. However, the percentage of patients indeed benefiting from adjuvant IFNα therapy is limited. Consequently, ever since the first reports demonstrating anti-tumor effects of IFNα for melanoma (5), several studies have been conducted to identify the optimal therapeutic schedule and the benefiting patient subpopulation. Meta-analyses of these trials demonstrated that IFNα has a consistent effect on relapse-free survival but no or only marginal effect on overall survival (OS), which had been attributed to the fact that only a subpopulation of patients benefit from treatment. A recent meta-analysis of two EORTC trials confirmed that limited tumor burden in stage III and ulceration of the primary tumor were the only predictive factors for adjuvant IFNα therapy (6).

Recent observations in other cancers suggest that the efficacy of IFNα therapy depends on the genetic predisposition. A screen of 463 single nucleotide polymorphisms (SNPs) in 33 candidate genes in metastatic renal cell carcinoma patients receiving IFNα therapy demonstrated a significant association of the *signal transducer and activator of transcription 3* (*STAT3*) SNP rs4796793 with clinical response (7). STAT3 is an integral molecule of the IFNα receptor signaling (8) and the rs4796793 SNP correlated with *STAT3* mRNA expression (7). This SNP is located in the 5′ region of the gene, 1633 bp upstream of the ATG site. In addition, in a murine melanoma model, blockade of STAT3 enhanced the therapeutic efficacy of IFN-alpha immunotherapy (9). These observations prompted us to scrutinize the impact of rs4796793 on the therapeutic efficacy of adjuvant IFNα in melanoma. Here, we report that despite the fact that there was no correlation between STAT3 mRNA expression and genotype, melanoma cells carrying the minor allele were more sensitive to IFNα *in vitro*. However, this notion did not translate into the clinical situation as the *STAT3* rs4796793 genotype did not correlate with the outcome of adjuvant IFNα treatment in stage III melanoma.

## Patients and Methods

### Genotyping

TaqMan allelic discrimination assay for SNP rs4796793 genotyping was purchased from Applied Biosystems (C27977213; Foster City, CA, USA). Polymerase chain reaction (PCR) was performed according to the manufactures instructions in 20 μl volume reactions with 1 μl DNA on a 7500 Fast Real time PCR system (Applied Biosystems).

### Quantitative RT-PCR analyses for STAT3

Endogeneous *STAT3* levels were determined for 35 peripheral blood lymphocytes (PBL) samples as well as 18 melanoma cell lines by real time PCR analyses in TaqMan technology using the comparative ΔΔ*C*_T_ method. PBL samples were obtained from melanoma patients who did not receive therapy at the time point the blood was drawn. Total RNA was isolated from approximately 3 × 10^6^ cells. Samples of total RNA were subjected to reverse transcription. Primers and probe for *STAT3* were designed with Primer Express software (Applied Biosystems, Weiterstadt, Germany). The assay (sense 5′-GGG CAC AAA CAC AAA AGT GAT G; antisense 5′-CAG CTC CTC AGT CAC AAT CAG G; probe 5′-FAM-AGA ATT CAA ACA CTT GAC CCT GAG GGA GCA) detects all three *STAT3* mRNA transcript variants. *GAPDH* (Applied Biosystems) served as endogenous control. The relative expression levels of *STAT3* normalized to *GAPDH* and relative to the PBL sample pat1 heterozygote for the SNP was calculated as 2^ΔΔ^*^C^*^T^ with ΔΔ*C*_T_ = (*C*_T STAT3, sample_ – *C*_T__GAPDH, sample_) – (*C*_T STAT3, pat1_ – *C*_T__GAPDH, pat1_). *C*_T_ is defined as the cycle when the threshold level of fluorescence is reached.

### Cell culture

Eleven melanoma cell lines were cultured in RPMI 1640 medium supplemented with 10% fetal calf serum. Four of these had the CC genotype (BLM, M19, M26, MelJuso), three the both genotype (FM79, FM82, Mel2A), and the remaining four the GG genotype (SkMel28, MaMel60, MaMel71, Mel888).

### MTS assay

In order to determine the impact of IFNα on melanoma cells, the MTS [3-(4,5-dimethylthiazol-2-yl)-5-(3-carboxymethoxyphenyl)-2-(4 sulfophenyl)-2H-tetrazolium] cell proliferation assay (Promega) was used according to the manufactures instructions. MTS is a tetrazolium reagent that is reduced by metabolically active cells. Melanoma cell lines were cultivated in triplicates in 96 well plates at 1000 (BLM, FM82, M26, Mel2A, Mel888, MelJuso, SKmel28), 4000 (FM79, M19, MaMel60), or 8000 (MaMel71) cells per well with normal medium or supplemented with 51,200 U/ml IFNα for 4 days. Extinction at 490 nm and background at 650 nm were measured with the Spectrostar^Nano^ (BMG Labtech, Ortenberg, Germany). First, with the blank corrected extinctions, the growth of the cells compared to the basal metabolic rate determined on day 1 before addition of IFNα was calculated. The inhibitory effect of IFN was then determined by (growth_medium control_ − growth_IFNα_)/growth_medium control_*100.

### Patients

Serum from advanced melanoma patients from frozen serum banks hosted by Skin Cancer Unit, Mannheim and the Department of Dermatology, Würzburg served as DNA source for genotyping as previously described (10). In order to be included into the study, the following criteria had to be fulfilled: (i) patients with histologically confirmed melanoma, (ii) a stage III diagnosis with a minimum follow-up of 2 months, (iii) Caucasian origin, and (iv) extended information available on their medical history including whether patients received adjuvant IFN therapy after stage III diagnosis. Patients with secondary malignancies were excluded from the study. Detailed patient characteristics are given in Table 1. The collection of sera and clinical data were performed after patients’ informed consent with Institutional Review Board approval. The presented work was conducted according to the principles expressed in the Declaration of Helsinki.

**Table 1:** **Patient characteristics**.

	All	No adjuvant therapy	Adjuvant IFNα therapy
**Gender**
F	119 (46.0%)	82 (44.8%)	37 (48.7%)
M	140 (54.1%)	101 (55.2%)	39 (51.3%)
Median age at diagnosis [IQR]	54.3 [41.4–64.7]	56.7 [43–65.5]	48.9 [35.9–61.6]
**Histological type**
ALM	26 (10.0%)	20 (10.9%)	6 (7.9%)
LMM	5 (1.9%)	5 (2.7%)	0
NM	94 (36.3%)	66 (36.1%)	28 (36.8%)
SSM	70 (27.0%)	49 (26.8%)	21 (27.6%)
Other	7 (2.7%)	5 (2.7%)	2 (2.6%)
Non-classifiable	7 (2.7%)	7 (3.8%)	0
Unknown	50 (19.3%)	31 (16.9%)	19 (25.0%)
**Ulceration pT**
yes	57 (22.0%)	43 (23.5%)	14 (18.4%)
no	109 (42.1%)	80 (43.7%)	29 (38.2%)
unknown	93 (35.9%)	60 (32.8%)	33 (43.4%)
***STAT3*** *rs4796793*
CC	135 (52.1%)	99 (54.1%)	36 (47.4%)
Both	102 (39.4%)	68 (37.2%)	34 (44.7%)
GG	22 (8.5%)	16 (8.7%)	6 (7.9%)

*ALM, acral-lentiginuous melanoma; LMM, lentigo maligna melanoma; IQR, inter- quartile range; NM, nodular melanoma; SSM, superficial spreading melanoma*.

### Statistical methods

Statistical analysis was performed with Prism (GraphPad La Jolla, CA, USA) or Stata 11.2 (StataCorp LP, College Station, TX, USA). For univariate analyses, the Kaplan–Meier method was used to compare survival time between groups. Differences of survival time were assessed by the log-rank test. IFNα sensitivity or *STAT3* expression of the different groups were compared by one-way ANOVA parametric when the data passed normality testing or else non-parametric, i.e., Kruskal–Wallis with Dunn’s post tests. Univariate as well as multivariate analyses Cox’s proportional-hazard regression model were applied when the models had passed the proportional-hazard assumption based on Schoenfeld residuals.

## Results

### *STAT3* rs4796793 genotype’s impact on *STAT3* mRNA expression and IFNα sensitivity

**Figure 1:**
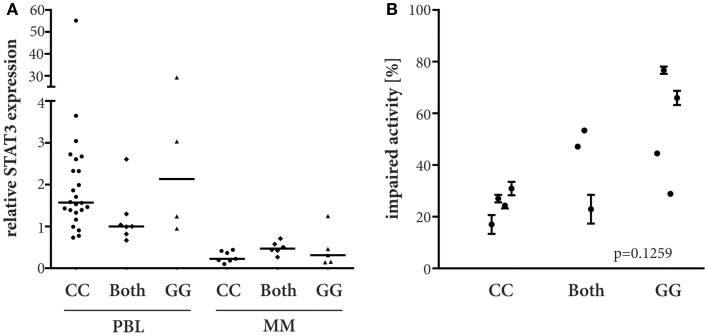
**STAT3 rs4796793 dependent endogeneous *STAT3* mRNA expression and IFNα sensitivity**. **(A)** *STAT3* mRNA expression was measured by real-time PCR in peripheral blood lymphocytes (PBL) and melanoma cell lines (MM). A CG genotype PBL sample served as calibrator. ANOVA analyses did not show significant differences (PBL, *p* = 0.0863, Kruskal–Wallis; MM, *p* = 0.3127, parametric). **(B)** MM lines were subjected to 51200 IU IFNα/ml or not, after 4 days their metabolic activity was measured by MTS assay. Depicted are the means with standard error for each cell line measured in at least two independent experiments.

It has been previously reported that the *STAT3* rs4796793 genotype correlates with endogenous *STAT3* expression in lymphocytes (7). To test the relevance of this observation in melanoma, particular in melanoma patients, we genotyped PBL and melanoma cell lines for *STAT3* rs4796793 SNP and subsequently measured the *STAT3* mRNA expression these. As expected from the role of STAT3 for lymphocytes, its expression was significantly higher in PBLs than in the melanoma cell lines (*p* < 0.0001; Kruskal–Wallis). However, within the two cell types, we could not detect a relevant difference of expression based on the SNP genotype (Figure 1A). Nevertheless, we next established if the *STAT3* rs4796793 was associated with IFNα sensitivity of melanoma cell lines. This analysis revealed a clear trend toward an increased IFNα sensitivity of melanoma cell lines with a homozygote *STAT3* rs4796793 minor allele. Indeed, the IFNα sensitivity increased from homozygote major allele to heterozygote and to homozygote with minor allele. This difference, however, was statistically not significant (*p* = 0.1259, Kruskal–Wallis; Figure 1B).

### *STAT3* rs4796793 genotype’s impact on the clinical course of melanoma

Two patient cohorts, i.e., with or without adjuvant IFNα therapy, were included to be able to distinguish if *STAT3* rs4796793 SNP is a predictive or a mere prognostic biomarker. Of the 259 patients, who were included, all had been diagnosed with or progressed to stage III melanoma. One hundred nineteen were female (46%) and 140 male (54.1%). The median age at diagnosis of stage III was 56.5 years. The median follow-up time from stage III diagnosis was 38.9 months; within this follow-up time, 159 patients developed distant metastases and 136 deaths were observed. About one-third (*n* = 76) of the patients had received IFNα as adjuvant therapy in stage III. Follow-up times for patients with or without adjuvant IFNα therapy were similar, but the treated cohort was significantly younger both at initial diagnosis or at progression to stage III (*p* = 0.008). Information on presence of absence of ulceration was available for 64.1% of the patients, i.e., 22% with, 42.1% without, and 35.9% with unknown ulceration status; these subpopulations were equally distributed among the two cohorts. Detailed patient and tumor characteristics and the genotypic frequencies of the *STAT3* rs4796793 SNP are given in Table 1. From all patients, the genotype could be determined. The observed genotype frequencies are similar to the frequencies reported for Europeans on the SNP database websites of the National Center for Biotechnology Information (dbSNP, http://www.ncbi.nlm.nih.gov/snp/) ranging from 54.2 to 58.3% for CC, 33.6 to 39% for CG, and 3.3 to 8.8% for GG.

**Figure 2:**
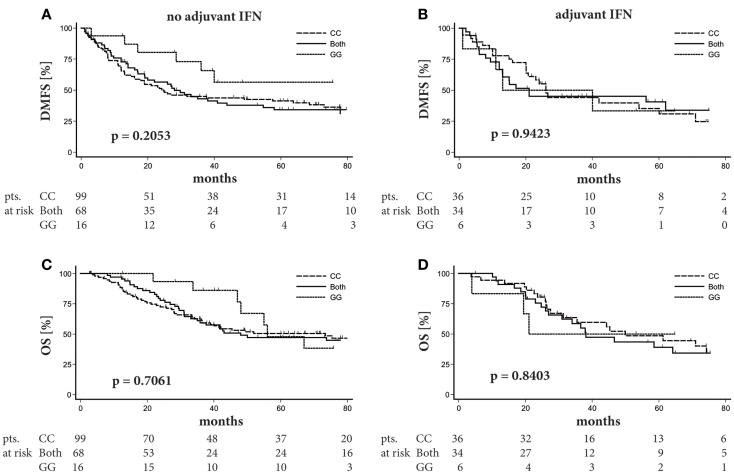
***STAT3* SNP rs4796793 does not influence distant metastasis free survival (DMFS) nor overall survival (OS) in stage III melanoma patients**. Patients were stratified according to their rs4796793 SNP. Kaplan–Meier plots for DMFS or OS of melanoma patients without **(A,C)** or with adjuvant IFNα therapy **(B,D)** in stage III. Below each graph, the patient numbers (pts.) at risk are given. Log-rank test was performed for statistical analysis.

Since therapeutic efficacy of adjuvant IFNα therapy is most evident in delay of disease progression, we analyzed the impact of rs4796793 on distant metastasis free survival (DMFS). For both the no-adjuvant-therapy group as well as the IFNα−adjuvant-therapy group, Kaplan–Meier analyses did not reveal any significant association between *STAT3* rs4796793 SNP genotype and DMFS (Figures 2A,B; *p* = 0.2053 or *p* = 0.9423, respectively; log-rank test). Despite the observation that the SNP genotype had no influence on DMFS in our patient groups, we tested for a potential effect of the *STAT3* rs4796793 SNP genotype on OS by the Kaplan–Meier method. Again, the rs4796793 genotype had no impact on survival for patients receiving IFNα adjuvant therapy or not (*p* = 0.8403 or *p* = 0.7061, respectively; log-rank test; Figures 2C,D). It should be further noticed that when we performed multivariate Cox regression analyses to adjust for gender and age at diagnosis of stage III, the SNP genotype still was not associated with the risk of progression. This was also the case when ulceration of the primary tumor was included in the analyses, but this information was available only for 64.1% of our patients reducing the size of the patient groups for analysis accordingly (data not shown).

## Discussion

Melanoma is regarded as one of the most lethal skin cancers. This is still true, despite major breakthroughs in melanoma research resulting in new therapies, such as small molecule kinase inhibitors or immune checkpoint blocking antibodies, which have been proven to be both effective and beneficial in advanced melanoma patients (11). Nevertheless, most patients responding to kinase inhibitors develop resistance to these and at best half of the patients respond to checkpoint blocking antibodies; thus, there is still an indication for adjuvant therapy for high-risk melanoma patients to avoid progression to metastatic disease. The only approved adjuvant therapeutic to date is IFNα. Unfortunately, a recent meta-analysis of 14 randomized clinical trials could neither identify an optimal IFNα dose and/or treatment duration nor the subset of patients benefiting from therapy (12). The latter is of particular importance as adjuvant IFNα therapy is associated with substantial toxicity in most of the patients (13). Consequently, a predictive biomarker would improve the risk (=toxicity) to benefit (=reduced risk of relapse/progression) ratio. In this regard, a recent meta-analysis of two EORTC trials demonstrated that only ulceration of the primary tumor and tumor stage are possible predictive factors for adjuvant IFNα (6).

Clinical efficacy of a therapeutic intervention, however, does not only dependent on tumor characteristics, but is also influenced by the patients’ genetics, which is particularly true for immune modulating therapies, such as IFNα (14). Notably, the effects of type I IFNs on the adaptive immune system are tightly regulated (15). One of the integral molecules of the IFN receptor signaling is STAT3 (8) and a SNP in S*TAT3* (i.e., rs4796793) has been reported to be associated with response to IFNα therapy in renal cell carcinoma patients (7). SNPs associated with response to therapy may affect the response either directly or indirectly by being in linkage disequilibrium with other disease-modulating alleles. In the case of rs4796793 and the effect of IFNα in renal cell carcinoma, it is assumed to be a direct effect, since the authors reported a small (*R*^2^ = 0.14) but significant correlation between rs4796793 and *STAT3* mRNA expression in Epstein–Barr virus transformed B-lymphocyte cell lines, i.e., a higher expression in cells being homozygote for the major allele (7). The increased endogeneous expression of *STAT3* mRNA in cell lines harboring the major allele of rs4796793 was explained by increased binding of NKX2-5 to the *STAT3* promoter, since this genotype contains an additional NKX2-5 binding site. Here, however, we did not observe a significant difference in endogeneous *STAT3* mRNA for PBLs or for melanoma cell lines depending on the *STAT3* rs4796793 genotype. These cell types may lack the respective transcription factor network, i.e., NKX2-5 and the antagonizing transcription factors NR2F1 and HMX1, or other transcription factors might be more relevant for endogenous *STAT3* expression in these cells.

Originally described for their antiviral activity, IFNα subtypes have demonstrated anti-tumor activity in different cancers (16). The therapeutic effect can be subdivided into those directly affecting tumor cells and those, which require immune mechanisms. IFNα exerts multiple biological effects, such as induction of apoptosis and inhibition of cell growth (17). The ability to enhance immune recognition of tumor cells by increasing MHC class I molecule expression resembles the intersection between direct and indirect effects of IFNα. The latter comprises several effects on immune competent host cells, e.g., enhancing differentiation of Th1 T-cell responses, generation, and activation of cytotoxic T–cells, as well as differentiation of DCs (16). Preclinical studies suggest that the applied dose determines which anti-tumor effect is triggered (18). In the adjuvant setting, direct effects on tumor cells are difficult – if not impossible – to determine, but the immune modulatory functions of IFNα are clearly evident. For example, in melanoma patients, the appearance of autoantibodies or clinical manifestations of autoimmunity during therapy was associated with an improved DMFS (19). Similarly, a therapy-associated increase of STAT1 activation in PBLs also correlated with the clinical benefit (20).

It is well established that IFN mediates its effect by STAT signaling (16). For example, the interferon-stimulated response elements (ISRE) of interferon-stimulated genes bind protein complexes containing phosphorylated STAT1 and STAT2 (21). Furthermore, it has been demonstrated that STAT3 expression increased the sensitivity to type I interferons in otherwise resistant cell lines (8). STAT3 has also been implicated in the clinical outcome of IFNα therapy. In a small study with 24 patients, the effect of IFNα treatment on phosphorylation of tyrosine at position 705 of STAT3 was determined (22). Phosphorylation of tyrosine 705 is necessary for STAT3 dimerization through phosphotyrosine-SH2 domain, which is a prerequisite for STAT3 transcriptional activity [reviewed in Ref. (23)]. Notably, STAT3 tyrosine 705 phosphorylation status in response to IFNα administration correlated with DMFS and OS. Since type I IFNs are weak activators of STAT3 (24), IFNα sensitivity should already be affected by the STAT3 expression level. Indeed, knockdown of STAT3 enhances IFNα mediated cell growth inhibition *in vitro*. These observations suggest that direct effect of the *STAT3* rs4796793 SNP are responsible for the reported effects on efficacy of IFNα in renal cell carcinoma patients (7). In a recent report by Kreil et al., the association between the *STAT5B* rs6503691 SNP and response of chronic myeloid leukemia (CML) to IFNα was demonstrated (25). Interestingly, this SNP was not related to *STAT5A* or *STAT5B* mRNA expression, but to *STAT3* mRNA expression, suggesting an association of *STAT3* expression and IFNα efficacy.

Although we did not detect a correlation between the *STAT3* rs4796793 genotype and *STAT3* mRNA levels, our *in vitro* results suggest that the presence of the minor allele increases the sensitivity of melanoma cells toward IFNα. Nevertheless, this *in vitro* effect did not translate into a clinical association. In the tested melanoma cohort receiving adjuvant IFNα therapy, no correlation between the *STAT3* rs4796793 genotype and DMFS or OS was evident. It should be noted that given the number of patients and the event rate in the IFNα treated patients, a Cox regression for DMFS achieves 80% power at a 0.05 significance level to detect a hazard ratio of 1.51. Thus, it is rather unlikely that if the *STAT3* rs4796793 SNP has a relevant impact, it would have been missed because of the size of the analyzed cohort. Consequently, the here presented data do not support a significant impact of the *STAT3* rs4796793 SNP on IFNα efficacy in melanoma patients.

## Conflict of Interest Statement

The authors declare that the research was conducted in the absence of any commercial or financial relationships that could be construed as a potential conflict of interest.

## Abbreviations

IFNα, interferon alpha; PBL, peripheral blood lymphocytes; STAT3, signal transducer and activator of transcription 3; SNP, single-nucleotide polymorphism.
